# Antibiotic use in childhood alters the gut microbiota and predisposes to overweight

**DOI:** 10.15698/mic2016.07.514

**Published:** 2016-06-20

**Authors:** Katri Korpela, Willem M de Vos

**Affiliations:** 1 Department of Bacteriology and Immunology, Immunobiology Research Program, University of Helsinki, Finland.

**Keywords:** microbiota, antibiotics, obesity, asthma, bile salt hydrolase, amoxicillin, macrolide, child

## Abstract

A correlation between the use of antibiotics in early life and the excessive weight gain in later childhood has been shown in several large cohort studies all over the world. One hypothesis explaining this association is the pervasive impact that antibiotics may have on the intestinal microbiota, and this has been supported by recent mouse studies. Studies have shown dramatic changes in the intestinal microbiota of adults in response to oral antibiotic treatments. However, little is known about the impact of antibiotics on the intestinal microbiota of children, although antibiotics account for the majority of the medication prescribed to children in Western countries.

In production animals antibiotic use increases weight gain at least partly by suppressing subclinical infections. In laboratory mice, living in clean and infection-free conditions, the antibiotic-induced weight gain was recently demonstrated by the group of Martin Blaser in New York, USA to be associated with an altered gut microbiome. Epidemiological studies have confirmed the positive relationship between antibiotic use and weight gain in humans and indicated that even prenatal antibiotic exposure predisposes to childhood overweight. Pre- and perinatal maternal and environmental factors are being recognized as important contributors to the long-term metabolic programming and weight development of infants, and multiple lines of evidence indicate that childhood overweight may be strongly dependent on early-life exposures. The intestinal microbiota, acquired initially during birth from the mother and nurtured by breast milk, are emerging as an important modulator of early metabolic programming, with long-lasting health consequences.

Our recent study addressed the association between antibiotic use and intestinal microbiota composition in healthy children. We studied a well-controlled cohort of 142 day care-attending children aged 2-7 years, collected data on their antibiotic use and health parameters, and coupled these too deep and global analysis of the intestinal microbiota and their functions. The microbiota composition in children exposed to penicillin or macrolide–type antibiotics during the previous 2 years was compared to the composition in children with no recent antibiotics and low lifetime antibiotic use. Our results confirmed a strong association between lifetime antibiotic use and BMI in children, and refined this observation by showing that the effect was evident only when the child had received macrolide antibiotics in early life. Remarkably, a similar strong effect of macrolides was detected on the intestinal microbiota. The most dramatic apparent effect of antibiotic treatments was a shift in the relative abundance of *Bifidobacterium* and *Bacteroides* after a macrolide course, which normalized within 24 months. In addition, a likely causal relation between the use of macrolide antibiotics and macrolide resistance was established, both at the genetic and phenotypic levels. Overall, macrolide use was associated with changes in the microbiome that have previously been linked with increased BMI, adiposity, obesity or metabolic diseases in children or adults: low overall richness, reduction of bacterial bile-salt metabolism, and reduction in bifidobacteria and *Christensenellaceae*, as well as increase in *Bacteroides* and *Erysipelotrichaceae*.

Earlier animal experiments of the Alimentary Pharmabiotic Centre in Cork, Ireland have shown that increasing the level of bile salt hydrolase decreased diet-induced obesity in mice. Further support to the antibiotic-induced disruption of bile-salt metabolism comes from a study by the group of Brett Finlay in Vancouver, Canada showing a reduction in bile-salt metabolism in mice experimentally treated with streptomycin. The microbiota changes associated with macrolide treatment may explain the obesogenic effect of early-life broad-spectrum antibiotic courses. Moreover, we found a number of bacterial taxa associated with high BMI: a combination of four genera differentiated obese vs. age-matched normal-weight children (Fig. 1). These included several bacterial groups that had been implicated in obesity development, such as a decreased level of *Akkermansia* spp., the dedicated mucus degrader that had earlier been discovered by our colleagues at Wageningen University, The Netherlands and protects mice from diet-induced obesity.

**Figure 1:**
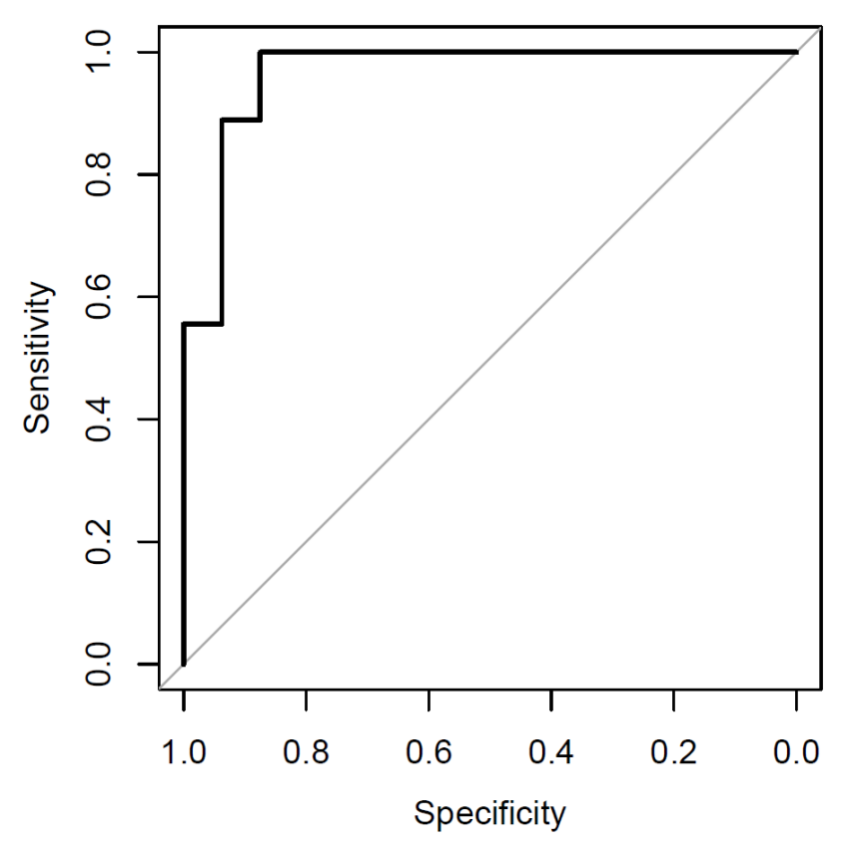
FIGURE 1: ROC analysis of the obesity prediction based on the abundance of four bacterial genera: Clostridium (*Clostridiaceae*), Clostridium (*Erysipelotrichaceae*), *Enterococcus*, and *Akkermansia*. AUC = 0.97.

Our results, supported by earlier experiments with mice suggest that the early-life microbiota is integrally involved in the long-term metabolic programming of infants. Increasing evidence is implying that bifidobacteria may have a key role in the metabolic programming of children. Finnish studies by the group of Erika Isolauri at Turku, Finland have noted a negative association between abundance of *Bifidobacterium* spp. in infancy and later BMI development. In adult humans, obesity and related metabolic markers have been shown to correlate negatively with the abundance of bifidobacteria. Work by the group of Nathalie Delzenne at Brussels, Belgium has shown that in rodents and to some extent in humans, bifidobacteria protect against diet-induced obesity and related metabolic effects. The beneficial metabolic effects of these species are thought to arise at least partly from improved gut barrier function, which reduces metabolic endotoxemia, i.e., abundance of bacterially produced LPS in the circulation. Circulating LPS induces inflammation, insulin resistance, and weight gain, and is considered an important component in the development of obesity and the related conditions. Bifidobacteria reduce the leakage of LPS from the gut presumably by up-regulating tight-junction proteins and can thereby improve the metabolic health of the host and counter-act diet-induced weight gain. Our study showed that macrolide treatment was associated with a reversion of the *Bifidobacterium*-to-*Bacteroides* balance, thus increasing the abundance of LPS-producing organisms and decreasing the abundance of gut-barrier-improving organisms. Frequent macrolide use may therefore involve recurrent LPS surges, similar to the effects of a high-fat diet, which could contribute to the association between antibiotic use and weight gain (Fig. 2). A full recovery of the microbiota from an antibiotic course appeared to take longer than the average interval between courses, which suggests that many children may not fully recover from antibiotic disturbance, but are in a continuously disrupted state throughout their early life (Fig. 2). Whether this has any permanent effects on the microbiota composition or function is currently not known, but mouse studies indicate that even temporary disruption of the microbiota in early life has long-term metabolic consequences.

**Figure 2:**
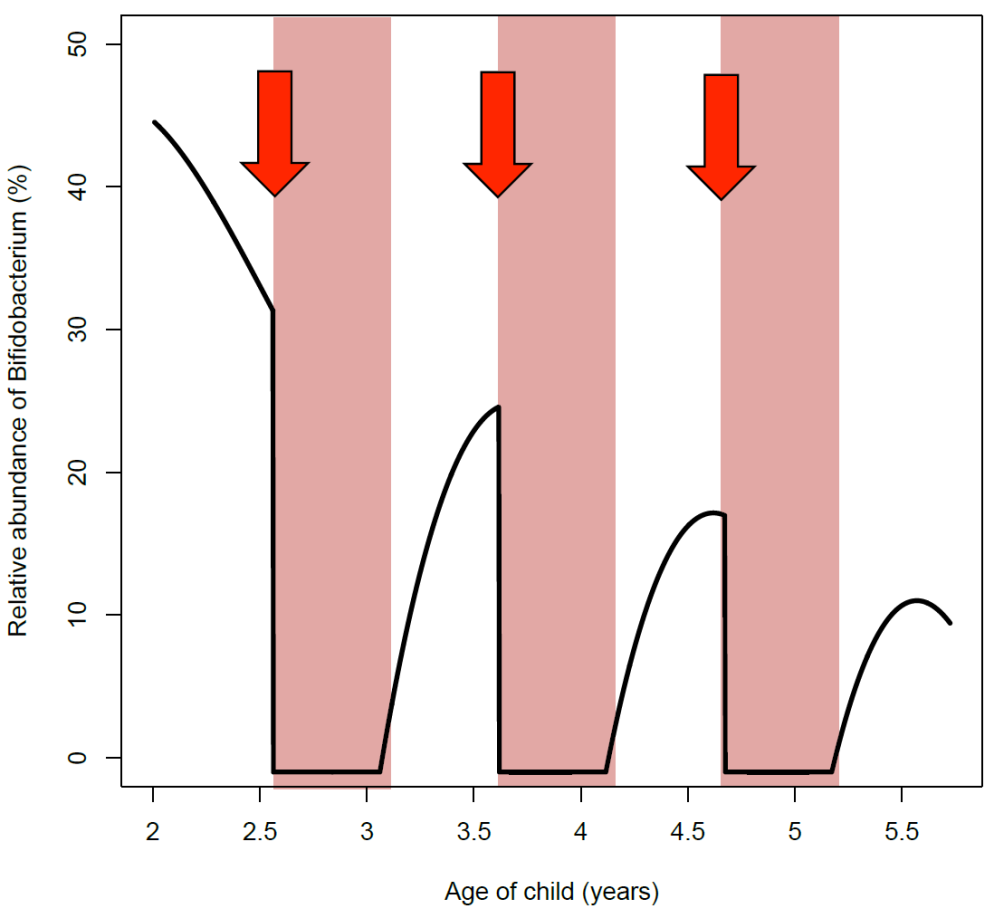
FIGURE 2: Schematic time course of *Bifidobacterium* abundance from the age of 2 years to 6 years. The abundance of *Bifidobacterium* naturally declines at this age as the microbiota mature, but macrolide courses (red arrows) cause a transient loss of *Bifidobacterium*, which is replaced by LPS-producing Gram-negative bacteria. This may result in the weakening of intestinal barrier, potentially causing increased translocation of LPS into the circulation (pink zones).

While antibiotics should be used when needed, our study indicates that it is important to consider the pervasive effects that macrolide use has on the microbiota as compared to that of penicillin-type antibiotics. Moreover, our present work provides further support in human for the hypothesis that the intestinal microbiota are involved in antibiotic-induced obesity and hence opens avenues to detect this in early stages and provide corrective therapies that target the intestinal bacteria.

